# Supplementary material for: Drinking Molecular Hydrogen Water Is Beneficial to Cardiovascular Function in Diet-Induced Obesity Mice
Source: Biology (Basel). 2021 Apr 23;10(5):364. doi: 10.3390/biology10050364 (PMC8146054; doi:10.3390/biology10050364)
Supplement: Supplementary file 1 [file biology-10-00364-s001.zip › biology-1151982-supplementary.pdf]

Drinking Molecular Hydrogen Water is Beneficial to Cardiovascular Function in Diet-Induced Obesity Mice

Haruchika Masuda [1,*], Atsuko Sato [1], Kumiko Miyata [1], Tomoko Shizuno [1], Akira Oyamada [2], Kazuo Ishiwata [1], Yoshihiro Nakagawa [3] and Takayuki Asahara [2]

Supplemental Material

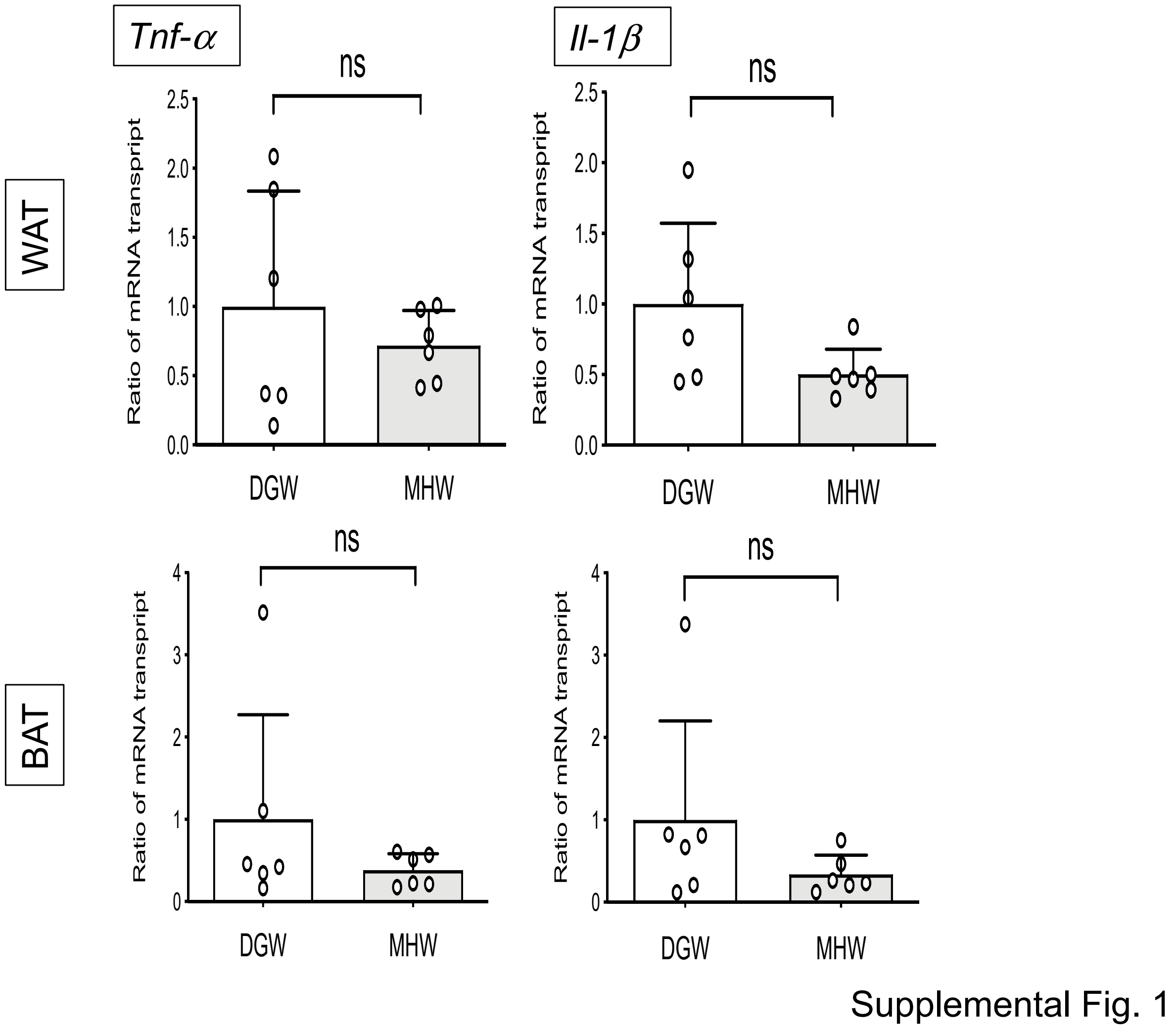

Supplemental Fig. 1. The genes expressing in BAT of DIO mice drinking MHW vs DGW for 2weeks.
Y axis indicates the transcriptional ratio of expressinig genes (*iNos, Tnf-α or Il-1β*) in WAT or BAT of DIO mice drinking MHW, compared to DGW. The bar graphs show mean ± SD.

Supplemental Table 1. The list of Taqman PCR probes for quantitative RT-PCR analysis.

Gene	Cat. No.	Co.
iNos	Mm00440502_m1	Applied Biosystems
eNos	Mm00435217_m1	Applied Biosystems
Vegf	Mm01281449_m1	Applied Biosystems
Ucp-1	Mm01244861_m1	Applied Biosystems
Tnf-α	Mm00443260_g1	Applied Biosystems
Il-1β	Mm00434228_m1	Applied Biosystems
18SrRna	Mm03928990_g1	Applied Biosystems

Supplemental Table 2. pH values of DGW or MHW.

	pre DGW	post DGW	pre MHW	post MHW
pH	8.1 ± 0.04	8.3 ± 0.44	8.9± 0.93	9.6 ± 0.96

n= 4 per group.

Supplemental Table 3. The changes of BW and PPBS in DIO mice drinking DGW or MHW for 14 days.

	BW (g)			PPBS (mg/dL)		
	day 0	day 7	day 14	day 0	day 7	day 14
DGW	29.1 ± 2.0	31.0 ± 2.3*	32.7 ± 2.4***	272.4 ± 57.5	246.6 ± 37.4	267.3 ± 46.3
MHW	30.3 ± 1.6	32.3 ± 1.5*	33.7 ± 1.6***	279.3 ± 46.3	260.7 ± 48.5	232.8 ± 45.6*

Asterisks indicate the statistical significance of the values at each time point versus day 0 in either of DGW or MHW. *$P < 0.05$, ***$P < 0.001$. n= 12.

Supplemental Table 4. The comparison of 8-OHdG+/8-OHdG- cells in WAT between MHW and DGW drinking DIO mice.

	DGW	MHW	*P* value
8-OHdG+ cells/mm^2	43.5 ± 10.5	17.4 ± 6.6	*0.0159**
8-OHdG- cells/mm^2	3.75 ± 2.5	11.6 ± 3.4	*0.0238**
Total cells/mm^2	47.3 ± 8.4	29.0 ± 8.6	*0.0317**

P values with asterisks indicate the statistical significance of MHW versus DGW analysed by Mann-Whiteny test. *$P < 0.05$. n= 4.

Supplemental Table 5. The ratios of genes expressing in adipose tissues in BAT or WAT of DIO mice drinking MHW or DGW.

	WAT			BAT		
Gene	DGW	MHW	*P* value	DGW	MHW	*P* value
iNos	1.0 ± 0.70	1.31 ± 0.54	0.3939	1.0 ± 0.82	1.64 ± 0.89	0.1255
eNos	1.0 ± 0.21	1.98 ± 0.95	*0.0411**	1.0 ± 0.16	1.64 ± 0.15	*0.0022**
Vegf	1.0 ± 0.38	1.29 ± 0.26	0.132	1.0 ± 0.28	1.57 ± 0.42	*0.026**
Ucp-1	1.0 ± 1.02	1.47 ± 1.62	0.8857	1.0 ± 0 .51	1.63 ± 0.30	*0.0317**
Tnf-α	1.0 ± 0.84	0.72 ± 0.26	>0.9999	1.0 ± 1.27	0.38 ± 0.20	0.6753
Il-1β	1.0 ± 0.57	0.50 ± 0.18	0.132	1.0 ± 1.20	0.34 ± 0.23	0.3095

P values with asterisks indicate the statistical significance of MHW versus DGW analysed by Mann-Whiteny test.

Supplemental Table 6. The comparison of 8-OHdG+/8-OHdG- cells in heart between MHW and DGW drinking DIO mice.

	DGW	MHW	*P* value
8-OHdG+ cells/mm^2	3,021 ± 1280	1,977 ± 361	0.1143
8-OHdG- cells/mm^2	124 ± 119	620 ± 307	*0.0286**
Total cells/mm^2	3,145 ± 1220	2,597 ± 652	0.6885

P values with asterisks indicate the statistical significance of MHW versus DGW analysed by Mann-Whiteny test. *$P < 0.05$. n= 4.

Supplemental Table 7. The cardiac function evaluated by echocardiography in DIO mice drinking MHW or DGW.

	DGW	MHW	*P* value
HR	237 ± 55	252 ± 11	0.6857
LV mass/BW (mg/g)	4.57± 0.20	3.64 ± 0.54	*0.0286**
FS (%)	22.8 ± 2.53	40.1 ± 5.54	*0.0286**
EF (%)	75.3 ± 4.4	90.3 ± 4.1	*0.0286**

Italic P values with asterisks indicate the statistical significance of MHW versus DGW analysed by Mann-Whiteny test.

Supplemental Table 8. The counts of EPC-CFUs in DIO mice drinking MHW or DGW.

	DGW	MHW	*P* value
PEPC-CFUs/mL blood	10.6 ± 9.1	21.0 ± 10.2	*0.0303**
DEPC-CFUs/mL blood	4.2 ± 5.2	16.9 ± 8.2	*0.0317**
Total EPC-CFUs/mL blood	14.8 ± 14.2	37.0 ± 16.7	*0.0281**
PEPC-CFUs/500 BM-KSL cells	31.7 ± 3.0	33.4± 4.0	0.6162
DEPC-CFUs/500 BM-KSL cells	9.0 ± 2.8	12.8 ± 2.4	*0.0038***
Total EPC-CFUs/500 BM-KSL cells	40.7 ± 3.6	46.3 ± 1.5	*0.0097***
SEPC-CFUs/Limb	1583 ± 566	2011± 329	*0.0422**
DEPC-CFUs/Limb	443 ± 190	778 ± 193	*0.0009****
Total EPC-CFUs/Limb	2026 ± 684	2790 ± 466	*0.0111**

Italic P values with asterisks indicate the statistical significance of MHW versus DGW analysed by Mann-Whiteny test.
